# Brief lifestyle advice in cardiac care: an experimental study on message source and framing

**DOI:** 10.1007/s12471-023-01827-7

**Published:** 2023-11-09

**Authors:** Renée V. H. IJzerman, Rosalie van der Vaart, Linda D. Breeman, Inge van den Broek, Mike Keesman, Roderik A. Kraaijenhagen, Thomas Reijnders, Margo Weerts, Andrea W. M. Evers, Wilma J. M. Scholte op Reimer, Veronica R. Janssen, the BENEFIT consortium

**Affiliations:** 1 Health, Medical and Neuropsychology Unit, Leiden University, Leiden, The Netherlands; 2 Department of Cardiology, Amsterdam University Medical Centres, University of Amsterdam, Amsterdam, The Netherlands; 3 Harteraad, Den Haag, The Netherlands; 4 NIPED, Amsterdam, The Netherlands; 5 Vital10, Amsterdam, The Netherlands; 6 Verbinden en Vernieuwen, Weesp, The Netherlands; 7 Medical Delta, Healthy Society, Leiden University, Technical University Delft, Erasmus University, Leiden, Delft, Rotterdam, The Netherlands; 8 Research Group Chronic Diseases, HU University of Applied Sciences, Utrecht, The Netherlands; 9 Department of Cardiology, Leiden University Medical Centre, Leiden, The Netherlands

**Keywords:** Cardiac Rehabilitation, Secondary Prevention, Lifestyle, Intention, Very Brief Advice

## Abstract

**Objective:**

Communicating risk information and offering lifestyle advice are important goals in cardiac rehabilitation. However, the most effective way and the most effective source to communicate this information are not yet known. Therefore, we examined the effect of source (cardiologist, physiotherapist) and framing (gain, loss) of brief lifestyle advice on patients’ intention-to-change-lifestyle.

**Methods:**

In an online experimental study, 636 cardiac patients (40% female, 67 (10) yrs.) were randomly assigned to one of four textual vignettes. Effect of source and framing on intention-to-change-lifestyle (assessed using a 5-point Likert scale) was analysed using analysis of covariance (ANCOVA).

**Results:**

Patients expressed positive intention-to-change-lifestyle after receiving advice from the cardiologist (*M* = 4.1) and physiotherapist (*M* = 3.9). However, patients showed significantly higher intention-to-change-lifestyle after receiving advice from the cardiologist (0.58 [0.54–0.61]) when compared with the physiotherapist (0.52 [0.48–0.56]), (*F*[1,609] = 7.06, *P* = 0.01). Gain-framed and loss-framed advice appeared equally effective. However, communicating risks (loss) was remembered by only 9% of patients, whereas 89% remembered benefits (gain).

**Conclusions:**

Our study shows the value of cardiologists and physiotherapists communicating brief lifestyle advice, as cardiac patients expressed positive intention for lifestyle change after receiving advice, irrespective of framing. Lifestyle advice should include benefits due to better recall.

**Supplementary Information:**

The online version of this article (10.1007/s12471-023-01827-7) contains supplementary material, which is available to authorized users.

## What’s new?

- To understand mechanisms influencing effectiveness of brief lifestyle advice, we examined the effect of message source (cardiologist, physiotherapist) and message framing (gain, loss) of brief lifestyle advice on intention-to-change-lifestyle in cardiac patients.
- This study demonstrates the importance of communicating brief lifestyle advice, as patients showed positive intention-to-change-lifestyle after receiving advice from the cardiologist and physiotherapist. However, patients showed significantly higher intention after receiving advice communicated by the cardiologist.
- Higher adherence to brief lifestyle advice may arise if healthcare providers focus on communicating benefits in addition to risks, as positively framed information was remembered better.

## Introduction

Engaging in a healthy lifestyle is crucial for secondary prevention of cardiovascular disease (CVD) [1, 2]. The European Guidelines on Prevention of Cardiovascular Disease in Clinical Practice recommend addressing risk information before referring patients to lifestyle interventions [1]. Using brief lifestyle advice, healthcare providers in cardiac rehabilitation can address modifiable cardiovascular risk factors such as smoking, physical activity, diet, and alcohol consumption in 2–3 min during a single consultation [3]. However, up to 50% of patients consciously choose not to follow recommendations [1]. Therefore, understanding the mechanisms influencing the effectiveness of brief lifestyle advice is essential to increase its impact [4, 5].

Message source [6, 7] and message framing [8, 9] have proved to affect acceptance of brief lifestyle advice. Messages delivered by highly credible sources (e.g., medical professionals) are perceived as more persuasive than those from less credible sources (e.g., non-experts) [6]. In the Netherlands, CVD patients often receive brief lifestyle advice from their cardiologist during quarterly consultations and from their physiotherapist during cardiac rehabilitation (CR). These healthcare professionals are both perceived as highly credible but differ in terms of frequency and duration of patient contact. However, possible differences in the effectiveness of brief lifestyle advice communicated by different credible sources are still unknown. Moreover, people have proved to respond differently to lifestyle advice communicated in terms of risks (loss-frame) or benefits (gain-frame) [10]. For example, “Quitting smoking has a positive effect on energy, appearance, and sleep quality.” (gain-frame) and “Continuing to smoke increases the risk of cardiovascular disease.” (loss-frame). It is unclear, however, which message frame is most effective when conveyed by various credible sources in cardiac rehabilitation. Therefore, this study examined whether CVD patients differ in intention to change their lifestyle after receiving either gain-framed or loss-framed brief lifestyle advice communicated by either a cardiologist or physiotherapist.

## Methods

### Study design

This study was an online vignette experiment on patients’ intention-to-change-lifestyle, using a 2-by‑2 between-subjects design. Following recommendations on conducting vignette studies [11], four written vignettes were developed to represent variations in source (cardiologist, physiotherapist) and framing (gain, loss) of brief lifestyle advice. This study protocol was approved by the Psychology Research Ethics Committee of Leiden University (2020-06-05‑A.W.M. Evers-V1-2474).

### Patient population and recruitment

The study population consisted of members of the patient panel of Harteraad, the Dutch patient association for CVD, a voluntary database of approximately 2,600 CVD patients. Harteraad sent their panel members a survey invitation on 29 June 2020, with a two-week response period. The invitation described the study’s purpose, duration, participation procedure, data protection, and the survey link (2020 Qualtrics, Provo, UT). Informed consent was obtained on the first page of the survey. Inclusion criteria were Dutch-speaking adults (≥ 18 years old). With a priori power analysis (two-sided, *α* = 0.05, power = 0.80) [12], 128 participants were required to detect an effect of source and framing on intention-to-change-lifestyle with a small effect size (*f* = 0.25). To account for 10% of dropouts, we aimed for at least 141 participants in total.

### Survey

**Fig. 1:**
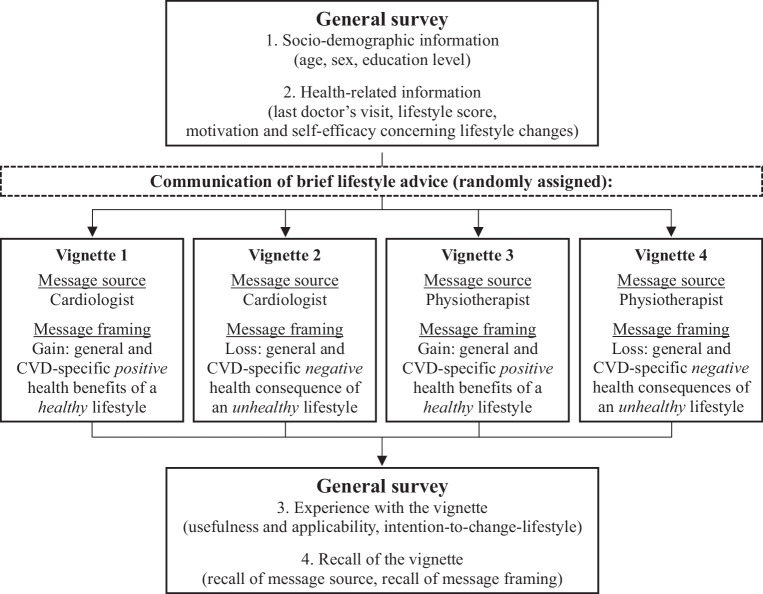
Design of the study. *CVD* cardiovascular disease

The survey first assessed patients’ characteristics (age, sex, education level, last doctor’s visit, current lifestyle score, motivation and self-efficacy concerning lifestyle change). Afterwards, patients were instructed to imagine having a consultation with their healthcare provider [11]. Each patient was then randomly assigned one of the four vignettes (see Fig. 1) which described brief lifestyle advice communicated by a cardiologist or physiotherapist (message source), using a gain-framed or loss-framed perspective on the health-related future (message framing). Vignettes (see App. A) had similar word counts, contained supporting images, and allowed reading the message aloud [11]. Afterwards, the vignettes’ applicability and meaningfulness were assessed, together with the primary outcome measure intention-to-change-lifestyle [13, 14]. Finally, used as manipulation check, patients were asked if they recalled which messenger delivered the brief lifestyle advice and what message was emphasised. After completing the survey, a debriefing explained the study’s purpose.

### Outcome measures

#### Main outcome

Similar to Taylor and colleagues (2005) [15], primary outcome measure intention-to-change-lifestyle was assessed using two items, “I want to change my lifestyle or continue to maintain my healthy lifestyle.” and “I intend to change my lifestyle or continue to maintain my healthy lifestyle.”, answered via a 5-point Likert scale, ranging from Completely disagree (1) to Completely agree (5) [16].

#### Other measures

Based on previous research [17], motivation, self-efficacy, and current lifestyle score were assessed with the questions “At this moment, how motivated do you feel to change your lifestyle or maintain your healthy lifestyle?”, “At this moment, how confident are you that you will be able to change your lifestyle or maintain your healthy lifestyle?”, and “At this moment, how would you rate your current lifestyle?”. Variables were rated on a 0–10 visual analogue scale (VAS) [18], representing a continuum between Very unmotivated/No confidence at all/Very unhealthy (0), and Very motivated/A lot of confidence/Very healthy (10). Perceived applicability and perceived meaningfulness of the vignette were assessed with the question, “Given your personal situation, how would you rate the conversation with the healthcare provider as just described?”. Variables were rated on a 0–10 VAS, representing a continuum between Not applicable at all/Not meaningful at all (0) and Very applicable/Very meaningful (10). Recall of message source was measured by asking “Which messenger were you speaking to?” with the answer options: Nurse, Cardiologist, Physiotherapist, or Mental health counsellor. Recall of message framing was measured by asking “What message did the healthcare provider emphasise?” with the answer options: a) “Positive effects of a healthy lifestyle” or b) “Negative consequences of an unhealthy lifestyle”.

### Statistical analysis

Data were analysed using IBM SPSS Statistics V.25. As the primary outcome intention-to-change-lifestyle was skewed, inverse transformation of intention was used. Two-way analysis of covariance (ANCOVA) examined the effect of source and framing on intention while controlling for sex, education level, motivation, and self-efficacy [19–22]. Bonferroni post hoc analyses were used for multiple comparisons. A *p*-value of < 0.05 was considered statistically significant.

Descriptive statistics were used for patient characteristics. Internal reliability was checked by calculating the scale score and Cronbach’s alpha for the intention scale, consisting of both intention items (*α* = 0.897). Pearson’s correlations were used to assess the strength and direction of the relationship between the control variables education level, motivation, and self-efficacy, and the dependent variable intention-to-change-lifestyle. An independent t‑test was used to assess the relationship between the control variable sex and dependent variable intention-to-change-lifestyle. Chi-squared tests were used to assess differences in recall between message source and types of message framing. Independent t‑tests were used to investigate differences in perceived applicability and meaningfulness of the vignettes for both sources and types of framing. Data are reported as *n *(%), mean (standard deviation), or mean (95% confidence interval).

## Results

### Patient characteristics

Tab. 1 describes characteristics of the 636 patients who completed the survey (estimated response rate: 24.5%). Of these patients, 383 were male (60%); mean age was 67 (10) years. Patients’ education levels were categorised as low (23%), medium (29%), high (47%). Intention-to-change-lifestyle was rated 3.9 (1.0) (range 1–5).

**Table 1:** Socio-demographic and health-related characteristics

Variable	Statistics
Age, years	67 (10)
Female sex	253 (40)
*Education level*	
Low (no education, and primary and preparatory vocational secondary education)	144 (23)
Medium (vocational, senior general secondary, and university preparation education)	185 (29)
High (higher vocational education and research university)	298 (47)
Other	9 (1)
*Last doctor’s visit*	
Past month	146 (23)
Past 2 to 6 months	238 (37)
More than 6 months ago	252 (40)
Current lifestyle score^a^	6.94 (1.75)
Motivation concerning lifestyle change^a^	6.89 (2.32)
Self-efficacy concerning lifestyle change^a^	6.88 (2.20)
Intention-to-change-lifestyle^b^	3.92 (1.00)

Data are *n *(%) or mean (standard deviation); mentioned when otherwise

^a^Question answered via a 0–10 visual analogue scale (low [0] to high [10])

^b^Statement answered via a 5-point Likert scale (Completely disagree [1] to Completely agree [5])

### Recall and vignette perceptions

After 35.5 (303) minutes on average, when the experiment ended, patients’ recall of source and framing was examined to determine the effectiveness of both manipulations. Significant associations were found between recall and source and between recall and framing, χ^2^(1) = 399.890, *P* = 0.05 and χ^2^(1) = 399.680, *P* < 0.001 respectively. Regarding source, 74% of the patients recalled the cardiologist, and 67% recalled the physiotherapist. Regarding framing, 89% of the patients recalled the gain frame, and 9% recalled the loss frame.

Concerning vignette perceptions (0–10 VAS), patients rated applicability 4.88 (2.69), and meaningfulness 5.22 (2.70). A significant difference in perceived meaningfulness between the cardiologist’s vignettes, 5.44 (2.63), and the physiotherapist’s, 4.99 (2.76), was found, *t* = 2.07, *P* = 0.04. No differences were found in other patient conditions.

### Effect of source and framing on intention-to-change-lifestyle

Tab. 2 presents the effect of message source and framing on intention-to-change-lifestyle. A two-way ANCOVA showed a significant main effect of source on intention, *F*(1,609) = 7.06, *P* = 0.01, while controlling for sex, education level, motivation, and self-efficacy. Patients expressed significantly higher intention after receiving advice from the cardiologist, 0.58 (0.54–0.61), than from the physiotherapist, 0.52 (0.48–0.56), *P* = 0.01. The main effect of framing on intention was non-significant, *F*(1,609) = 0.01, *P* = 0.92, as was the interaction effect between source and framing on intention, *F*(1,609) = 0.42, *P* = 0.52. Repeating the analysis without covariates showed similar results.

**Table 2:** Effect of message source and message framing on intention-to-change-lifestyle

			Effect of the manipulations			
	Adjusted value	Unadjusted value	*Adjusted* *F* (df = 609)^a^	*P*	*Unadjusted* *F* (df = 632)	*P*
*Message framing*			0.01	0.92	0.08	0.78
Gain	0.55 (0.51–0.59)	0.61 (0.58–0.64)				
Loss	0.45 (0.51–0.59)	0.60 (0.57–0.63)				
*Message source*			7.06	0.01	5.65	0.02
Cardiologist	0.58 (0.54–0.61)	0.63 (0.60–0.66)				
Physiotherapist	0.52 (0.48–0.56)	0.58 (0.55–0.61)				
*Message framing* *x message source*			0.42	0.52	0.04	0.84
Gain x cardiologist	0.57 (0.52–0.62)	0.63 (0.59–0.67)				
Gain x physiotherapist	0.53 (0.48–0.58)	0.58 (0.54–0.63)				
Loss x cardiologist	0.58 (0.53–0.63)	0.63 (0.58–0.67)				
Loss x physiotherapist	0.52 (0.47–0.56)	0.57 (0.53–0.61)				

Values are estimated mean (95% confidence interval)

^a^Adjusted for sex, education level, motivation, and self-efficacy

*df* degrees of freedom

## Discussion

This study demonstrates the value of communicating brief lifestyle advice to cardiac patients, as they expressed positive intentions to change their lifestyle after receiving advice from either the cardiologist or the physiotherapist. Generally, however, a higher intention to change was expressed when the advice was communicated by the cardiologist; this difference in message source was found to be significant. Gain-framed or loss-framed advice appeared equally effective for patients’ intention. However, gain-framed information was recalled better than loss-framed information.

Concerning message source, the fact that patients expressed a significantly higher intention after receiving advice from the cardiologist than the physiotherapist may be due to slightly different perceived credibility towards the healthcare providers. Possibly, patients make a subtle distinction between a cardiologist and physiotherapist when evaluating their expertise, trustworthiness, or caring [7]. Specifically, patients appear more likely to adhere to treatment advice when communicated by credible physicians [23] and feel more satisfied with the healthcare received when they perceive the source as competent or caring [24]. This may explain why patients rated the meaningfulness of the cardiologist’s vignette higher, even though it was identical to the physiotherapist’s vignette, and recalled the advice from the cardiologist better than the advice from the physiotherapist.

Concerning message framing, gain-framed and loss-framed brief lifestyle advice appeared equally effective on intention. This contrasts with prior research that showed a more positive influence of gain-framed health messages on patient evaluation of treatment advice and their intention to change preventive lifestyle behaviour [8, 9]. However, these studies mainly focussed on specific lifestyle behaviour (e.g., smoking cessation) rather than generic lifestyle behaviour as we did. Moreover, in line with our results, meta-analyses have shown that communicating either gain-framed or loss-framed advice did not have significantly different advantages for patients’ intentions to change lifestyle [10]. Nevertheless, patients rated the vignette’s applicability and meaningfulness as average, but this may have been different if it had been discussed in the consultation room and the advice had been tailored to the patient under the healthcare provider’s guidance.

Subsequently, patients recalled gain-framed advice more often than loss-framed advice. This may be due to patients undergoing treatment for an extended period and probably already receiving similar lifestyle advice. Moreover, patients’ memory representation may have affected memories of the received advice; exact details such as framing of words quickly become inaccessible because of rapid memory decline during the first 15 to 30 min after exposure to the message. After this period, an essential meaning of the message is remembered [25]. Finally, the positivity effect, which is the tendency for older adults to remember positive information and disregard negative information, may also have played a role in this outcome [26].

### Limitations and implications for practice

Concerning limitations, loss-framed lifestyle advice appeared challenging to recall. Possibly, the manipulation of message framing was not strong enough or insufficiently specified, or healthcare providers had already communicated the generic health-related information described in the vignette. As a result, message framing might not have been striking enough. Next, this study specifically explored the impact of source and framing on intention after providing generic brief lifestyle advice, using vignettes solely describing the advice. Future research could examine whether intention increases when vignettes convey the steps required to implement the desired lifestyle change. Furthermore, even though intention is an essential predictor of actual behaviour [27], there is a gap between intention and actual behaviour [28]. Additionally, absence of direct face-to-face consultations may have influenced our findings’ generalisability to real-world settings. While our vignettes were developed based on scientific recommendations [11] and aimed to simulate realistic patient-provider interactions in cardiac care, we emphasise the need for future research to validate our findings in actual clinical settings.

Current ESC guidelines on cardiovascular disease prevention recommend providing risk information to stimulate attendance to lifestyle interventions [1]. This study found that only 9% of patients remembered risk communication, whereas 89% remembered communication of benefits. To improve adherence to brief lifestyle advice, healthcare providers should emphasise the benefits of healthy living in addition to risks. Additionally, the mere endorsement of a healthcare provider may bolster patients’ intention-to-change-lifestyle. Given physicians’ limited consultation time, emphasising the value of a healthy lifestyle without extensive discussion of associated behaviours can be effective. Easy referral to other healthcare providers using motivational interviewing techniques [29, 30], for example, physiotherapists, generalist or specialist nurses, or healthcare providers working at lifestyle care counters, will further facilitate suitable treatment and sustainable lifestyle change.

## Conclusion

Cardiac patients expressed the highest intention to change their lifestyle when brief lifestyle advice was communicated by a cardiologist. This was irrespective of message framing, but communicating benefits rather than risks appeared to be related to better recall. The results and associated practical implications can support healthcare providers in cardiac care in optimising the brief lifestyle advice they provide their patients to facilitate suitable treatment and sustainable lifestyle change.

### Supplementary Information

Appendix A
