# Supplementary material for: Brief lifestyle advice in cardiac care: an experimental study on message source and framing
Source: Neth Heart J. 2023 Nov 9;32(1):38–44. doi: 10.1007/s12471-023-01827-7 (PMC10781907; doi:10.1007/s12471-023-01827-7)
Supplement: Supplementary file 1 — Appendix A [file 12471_2023_1827_MOESM1_ESM.docx]

**Appendix A**

Vignette 1: Cardiologist and Gain framing

**Because of your cardiovascular disease, your cardiologist invites you to discuss your lifestyle.**

The cardiologist greets you upon arrival, introduces themself, and asks how you are doing. The cardiologist
will then advise you on how to change your lifestyle or maintain your healthy lifestyle.


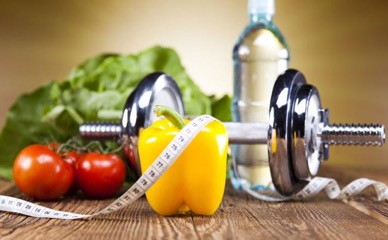
The cardiologist tells you that **a healthy lifestyle
ensures that you feel well, both mentally and physically. Moreover, healthy living habits can improve your cardiovascular condition.**

The cardiologist tells you that **everyone can achieve
some profit in terms of lifestyle, even if only a little**.
You can think of sufficient exercise, eating healthy,
sleeping well, dealing with stress or tension, quitting smoking, and reducing/quitting alcohol consumption.

With the cardiologist, you can determine what would make the most sense to get started with.
The cardiologist will be happy to refer you to a lifestyle program that suits your needs and personal goals.
You can discuss these next steps together at another time.

Vignette 2: Cardiologist and Loss framing

**Because of your cardiovascular disease, your cardiologist invites you to discuss your lifestyle.**

The cardiologist greets you upon arrival, introduces themself, and asks how you are doing. The cardiologist
will then advise you on how to change your lifestyle or maintain your healthy lifestyle.

The cardiologist tells you that **an unhealthy lifestyle
ensures that you feel worse, both mentally and physically. Moreover, unhealthy living habits can worsen your
cardiovascular condition.**


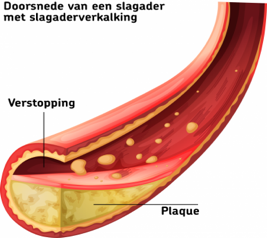


Blockage

**Cross-section of an artery with atherosclerosis**

Plaque

The cardiologist tells you that **everyone can achieve
some profit in terms of lifestyle, even if only a little**.
You can think of sufficient exercise, eating healthy,
sleeping well, dealing with stress or tension, quitting
smoking, and reducing/quitting alcohol consumption.

With the cardiologist, you can determine what would make the most sense to get started with.
The cardiologist will be happy to refer you to a lifestyle program that suits your needs and personal goals.
You can discuss these next steps together at another time.

Vignette 3: Physiotherapist and Gain framing

**Because of your cardiovascular disease, your physiotherapist invites you to discuss your lifestyle.**

The physiotherapist greets you upon arrival, introduces themself, and asks how you are doing. The physiotherapist will then advise you on how to change your lifestyle or maintain your healthy lifestyle.


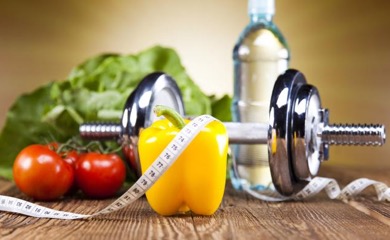
The physiotherapist tells you that **a healthy lifestyle ensures that you feel well, both mentally and physically. Moreover, healthy living habits can improve your cardiovascular condition.**

The physiotherapist tells you that **everyone can achieve some profit in terms of lifestyle, even if only a little**.
You can think of sufficient exercise, eating healthy,
sleeping well, dealing with stress or tension, quitting smoking, and reducing/quitting alcohol consumption.

With the physiotherapist, you can determine what would make the most sense to get started with.
The physiotherapist will be happy to refer you to a lifestyle program that suits your needs and personal goals.
You can discuss these next steps together at another time.

Vignette 4: Physiotherapist and Loss framing

**Because of your cardiovascular disease, your physiotherapist invites you to discuss your lifestyle.**

The physiotherapist greets you upon arrival, introduces themself, and asks how you are doing. The physiotherapist will then advise you on how to change your lifestyle or maintain your healthy lifestyle.


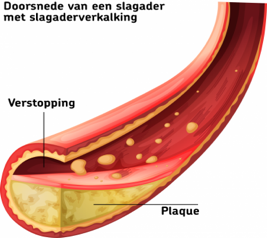


Blockage

**Cross-section of an artery with atherosclerosis**

Plaque

The physiotherapist tells you that **an unhealthy lifestyle
ensures that you feel worse, both mentally and physically.
Moreover, unhealthy living habits can worsen your
cardiovascular condition.**

The physiotherapist tells you that **everyone can achieve
some profit in terms of lifestyle, even if only a little**.
You can think of sufficient exercise, eating healthy,
sleeping well, dealing with stress or tension, quitting
smoking, and reducing/quitting alcohol consumption.

With the physiotherapist, you can determine what would make the most sense to get started with.
The physiotherapist will be happy to refer you to a lifestyle program that suits your needs and personal goals.
You can discuss these next steps together at another time.
